# Crisaborole as a Potential Treatment for Pediatric Alopecia Areata: A Case Report and Literature Review

**DOI:** 10.7759/cureus.102854

**Published:** 2026-02-02

**Authors:** Qiannan Xu

**Affiliations:** 1 Dermatology, Shanghai East International Medical Center, Shanghai, CHN

**Keywords:** alopecia areata (a.a.) treatment, crisaborole, hair regrowth, pediatric treatment, phosphodiesterase 4 (pde4) inhibitor

## Abstract

Alopecia areata (AA) is an autoimmune disorder that causes non-scarring hair loss, often affecting pediatric patients, where it can significantly impact both psychological well-being and quality of life. While corticosteroids and other systemic therapies are commonly used to treat AA, their side effects, especially in children, highlight the need for safer alternatives. This case report evaluates the use of crisaborole, a topical phosphodiesterase 4 (PDE4) inhibitor, as a potential treatment for pediatric AA. A 10-year-old female patient, who initially presented with a small patch of hair loss and failed to respond to topical corticosteroid therapy, was switched to crisaborole after concerns about corticosteroid-related side effects. After two weeks of treatment, significant hair regrowth was observed, and a follow-up at three months confirmed full regrowth. The mechanisms through which crisaborole may benefit AA include modulation of inflammation, improvement in blood flow to hair follicles, and promotion of follicular stem cell activity. Crisaborole's safety profile, with minimal systemic absorption, makes it a promising non-corticosteroidal alternative for pediatric patients. This case suggests that crisaborole may be an effective treatment for pediatric AA, but further studies with larger sample sizes and longer follow-up periods are needed to validate these findings.

## Introduction

Alopecia areata (AA) is a common autoimmune disorder characterized by non-scarring hair loss. AA affects approximately 2% [1] of the global population, with a higher incidence in children and young adults. Studies estimate that 20% of AA cases occur in children under 12 years, with a lifetime risk of 1.7-2.1%, which has a significant impact on the psychological well-being and quality of life, particularly in pediatric patients [2]. The pathogenesis of AA involves immune-mediated destruction of hair follicles, with CD8+ T cells playing a pivotal role in the inflammatory process [3]. Current treatments for AA include corticosteroids, topical immunotherapy, and systemic therapies such as Janus kinase (JAK) inhibitors. However, no FDA-approved treatments exist for pediatric AA patients under the age of 12, highlighting the urgent need for safer and more effective therapeutic options. When selecting a treatment plan for pediatric AA patients, factors such as the patient's age, disease severity, treatment tolerability, and potential side effects must be considered. Non-systemic treatments such as crisaborole may be preferred to minimize risks associated with systemic therapies, particularly in younger children with sensitive skin. This report explores the potential of crisaborole, a topical phosphodiesterase 4 (PDE4) inhibitor, as an alternative treatment for AA, presented in the context of a pediatric case where topical corticosteroid therapy failed.

## Case presentation

**Figure 1:**
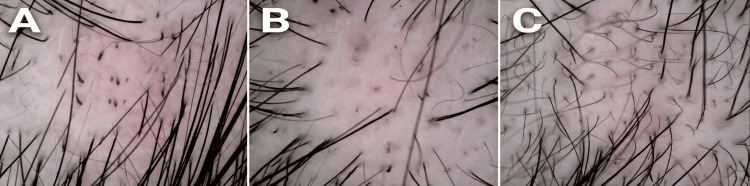
Microscopic Analysis of the Scalp Condition Following Treatment Over a Four-Week Period (A) Pre-treatment: Microscopic image of the scalp prior to treatment, exhibiting a high density of debris and signs of irritation, with hair follicles appearing sparse and irregularly distributed. (B) Two-Week Post-treatment: Microscopic image of the scalp after two weeks of treatment, demonstrating a marked reduction in debris and improved scalp clarity, with hair follicles showing slight enhancement in definition and distribution. (C) Four-Week Post-treatment: Microscopic image of the scalp after four weeks of treatment, revealing significant improvement characterized by a cleaner scalp surface, reduced irritation, and more uniform hair follicle distribution, suggesting an effective therapeutic response. In dermatoscopy for alopecia areata, black dots are remnants of fractured hair shafts at the follicular opening, appearing as small black specks representing destroyed or cadaverized hairs; they indicate active disease and rapid hair loss. Broken hairs include fragments of shafts broken at various lengths, such as exclamation mark hairs with a tapered base and normal tip, resulting from immune attacks on follicles and signaling progression. Newly grown hairs are short vellus hairs in recovery—fine, soft, lightly colored regrowth from follicles, often with white or upright hairs, denoting stabilization.

The patient was a 10-year-old female of Asian descent, with no prior personal or family history of AA or other autoimmune disorders. Her parents reported no associated medical conditions, such as thyroid disease, atopic dermatitis, or vitiligo, and denied any recent stressors, infections, or vaccinations that might have precipitated the onset. The patient presented to our dermatology clinic with a two-week history of localized hair loss on the scalp, which had been noticed incidentally by her mother during hair brushing. There were no accompanying symptoms, such as scalp pain, pruritus, scaling, or erythema, and the patient was otherwise healthy, with normal growth and development for her age. On physical examination, a single, well-defined, oval patch of alopecia measuring approximately 1 cm in diameter (fingernail-sized) was noted on the parietal scalp. The patch was smooth and non-scarring, with no signs of inflammation, scarring, or secondary infection. Surrounding hair appeared normal, and there were no exclamation mark hairs visible to the naked eye. No involvement of other body sites, such as eyebrows, eyelashes, or nails, was observed. Trichoscopy, performed using a handheld dermatoscope at 20x magnification, confirmed diagnostic features consistent with active AA, including multiple black dots (indicating dystrophic anagen hairs), broken hairs, and yellow dots (representing empty follicular ostia filled with sebum and keratin) (Figure 1A).

No taper hairs or cadaverized hairs were noted, and the pull test was negative in adjacent areas. Routine laboratory investigations, including complete blood count, thyroid function tests, and antinuclear antibody screening, were within normal limits, ruling out associated systemic autoimmune or endocrine disorders. No skin biopsy was performed, as the clinical and trichoscopic findings were characteristic of AA, and the lesion was limited in extent. The initial treatment plan involved topical mometasone furoate cream (0.1%, MSD) applied twice daily to the affected area, with the expectation of inducing hair regrowth within four to six weeks while monitoring for local side effects, such as skin atrophy or telangiectasia. This choice was based on standard guidelines for mild, localized pediatric AA, aiming for anti-inflammatory effects to halt disease progression. No oral medicine was given. However, at the two-week follow-up, the patient's mother reported non-adherence due to concerns over potential steroid-related adverse effects, including skin thinning and systemic absorption in a child, leading to reduced application to once daily. Re-examination revealed no evidence of hair regrowth; instead, the patch had slightly expanded to approximately 1.5 cm in diameter, with additional broken hairs noted on trichoscopy, indicating disease progression (Figure 1B).

Given the lack of response, parental anxiety regarding steroids, and the need for a safer alternative, the treatment was switched to topical crisaborole ointment (2%), applied twice daily to the affected patch. The expected outcome was a gradual reduction in inflammation and promotion of hair regrowth over four to eight weeks, with minimal risk of side effects due to its non-steroidal mechanism and low systemic absorption. The actual outcome exceeded expectations. After two weeks of crisaborole therapy, follow-up trichoscopy demonstrated significant improvement, including the emergence of new vellus hairs, reduced black dots, and fewer yellow dots, signifying early follicular recovery (Figure 1C). The patient tolerated the treatment well, with no reported adverse events, such as burning, stinging, or irritation. A subsequent online consultation at three months post-initiation confirmed complete hair regrowth in the affected area, with terminal hairs fully restoring the patch to match surrounding scalp density. No recurrence was noted at this point, and the patient was advised to continue monitoring for any new lesions.

## Discussion

Pearl of the case

Although AA can be self-limiting, complicating attribution of regrowth to crisaborole versus corticosteroid inefficacy, case-specific factors justify the switch. In real-world clinical practice, topical corticosteroids (e.g., mometasone) for AA do not show immediate effects, particularly in the progressive stage (as evidenced by persistent black dots, yellow dots, and broken hairs on trichoscopy in this case); literature indicates that the onset time is typically two to three months for initial hair regrowth, with a median time to significant regrowth of 12.2 months [3]. In this case, after only two weeks of steroid use, trichoscopy showed disease progression (patch expansion, new broken hairs), possibly due to upregulation of CCL20 expression: studies confirm that glucocorticoids promote CCL20 expression in human keratinocytes and murine skin, even in undisturbed states [4]. CCL20, a chemokine, recruits more immune cells (e.g., Th17 cells), exacerbating local inflammation and microenvironment damage, leading to short-term worsening rather than relief. This aligns with the case: while steroids may clear pathogenic T cells, CCL20 upregulation may synchronously cause treatment-related damage (e.g., long-term skin thinning, telangiectasia), delaying onset or aggravating disease.

Children's hair growth cycles differ from adults, generally faster: children's growth rate can reach 345 μm/day in three-year-olds, compared to adult averages of 0.3-0.4 mm/day [5]. Once effective, new hairs (vellus) can grow rapidly, restoring density in weeks, explaining the two-week new hair growth post-switch in this case. However, real-world data show that pediatric AA treatment is often more difficult than in adults [6]. This may be due to more active pathogenic immune cells in children, leading to severe microenvironment damage post-infiltration. Effective treatment requires improving both pathogenic cells and the microenvironment, easier in adults. Combining parental anxiety (self-reduced dosing leading to poor compliance), switching rather than adhering to guidelines (continuing corticosteroids) may be better: real-world data show adults start treatment 2.2 days post-diagnosis (with 2.6 for children, duration: 76.9 vs 64.3 days), reflecting adherence challenges [7]. Crisaborole, a non-corticosteroidal PDE4 inhibitor, alleviates guardian anxiety; its mechanism increases cAMP, inhibiting cytokines (TNF-α, IL-17) effective in atopic dermatitis (similar to AA, driven by pathogenic immune cells damaging microenvironment) [8]. In this case, we replaced corticosteroids with it. The unexpected two-week hair growth may be because crisaborole not only inhibits pathogenic cells but also regulates the microenvironment: literature shows that PDE4 inhibitors reduce proinflammatory mediators, and similar compounds lower CCL20 [9]. Prior corticosteroid-induced CCL20 may, under crisaborole, convert to a hair growth-promoting factor (e.g., improving vascularization).

Challenges in pediatric AA treatment

Treating AA in pediatric patients is challenging due to the lack of FDA-approved therapies for children under 12 years of age. Corticosteroid-based treatments, while effective, carry the risk of significant side effects, including growth retardation and skin thinning, particularly with long-term use. This case demonstrates the need for alternative therapies that can effectively treat AA while minimizing adverse effects, especially in the pediatric population. 

Crisaborole: A PDE4 inhibitor

Crisaborole is a topical PDE4 inhibitor approved for treating atopic dermatitis in children over the age of three months. PDE4 inhibitors work by increasing intracellular cyclic adenosine monophosphate (cAMP) levels, which in turn modulate immune responses and inflammation. This makes them a promising option for autoimmune diseases such as AA, where inflammation and immune dysregulation are key drivers of hair follicle destruction. PDE4 inhibitors, including crisaborole, modulate inflammatory responses by reducing pro-inflammatory cytokines, such as TNF-α and IL-17, which are elevated in AA. These mechanisms may improve disease outcomes by decreasing perifollicular inflammation and promoting hair regrowth.

Proposed mechanisms of crisaborole in AA treatment

The therapeutic benefits of crisaborole in AA may be explained by several mechanisms:

Inflammation modulation: Increased cAMP levels from PDE4 inhibition reduce the production of inflammatory cytokines, such as IL-4 and IL-17, which are involved in the autoimmune processes of AA [10-12]. This downregulation of pro-inflammatory mediators may help alleviate the immune attack on hair follicles.

Vascular effects: Elevated cAMP levels are associated with vasodilation, which could improve blood flow to hair follicles, thereby supporting the regrowth of hair [13]. Enhanced vascularization in the scalp might create a more favorable environment for hair follicle regeneration.

Stem cell activation: High cAMP concentrations have been linked to the activation and differentiation of follicular stem cells [14]. By enhancing stem cell activity, PDE4 inhibitors such as crisaborole may promote hair follicle regeneration and recovery.

Table 1 presents the mechanisms, effects, and clinical considerations of crisaborole in the treatment of AA.

**Table 1:** Mechanisms, Effects, and Clinical Considerations of Crisaborole in the Treatment of Alopecia Areata Table Credits: Dr. Qiannan Xu

Mechanism/Effect	Explanation	Relevance to Alopecia Areata (AA)
Inflammation Modulation	PDE4 inhibitors increase intracellular cAMP levels, which reduces the activation of pro-inflammatory cytokines like IL-4, IL-17, and TNF-α [15].	AA is an autoimmune disorder where inflammatory cytokines, especially IL-17, play a crucial role in attacking hair follicles. PDE4 inhibition can help reduce this immune response [16].
Vasodilation and Improved Circulation	PDE4 inhibition promotes vasodilation (relaxation of blood vessels) through increased cAMP, improving blood flow [17].	Better circulation may enhance oxygen and nutrient supply to hair follicles, promoting the regrowth of hair. AA often involves ischemia or reduced blood flow to affected hair follicles.
Reduction of Immune Cell Infiltration	By modulating cAMP levels, PDE4 inhibitors can decrease the migration and activation of immune cells, including T cells and macrophages [17].	AA involves infiltration of immune cells, particularly CD8+ T cells, into hair follicles, leading to their destruction. PDE4 inhibition may help prevent this infiltration.
Promotion of Follicle Stem Cell Activity	Elevated cAMP is linked to stem cell activation and differentiation. PDE4 inhibition may enhance follicle stem cell activity, aiding hair follicle regeneration [17].	Hair regrowth in AA is dependent on follicular stem cell activity. PDE4 inhibitors could promote the activation and differentiation of these stem cells to restore hair growth.
Anti-Itch and Skin Irritation Relief	PDE4 inhibitors like crisaborole have been shown to reduce pruritus (itching) and skin irritation by downregulating inflammatory mediators involved in itching [18].	Pruritus is a common symptom in AA, and a reduction in skin irritation could improve the patient's comfort during treatment, especially in pediatric cases.
Safer for Long-Term Use	Crisaborole has a favorable safety profile with minimal systemic absorption, making it a safer option for long-term use compared to topical corticosteroids [19].	Given the concerns about long-term corticosteroid use in pediatric patients, crisaborole offers a safe, non-corticosteroidal alternative for managing AA without the risk of systemic side effects.
Non-Systemic Absorption	Crisaborole, being a topical treatment, is minimally absorbed systemically, reducing the risk of systemic side effects such as growth retardation or adrenal suppression [20].	Corticosteroid treatments often carry risks of systemic absorption, especially in children. A topical PDE4 inhibitor provides targeted, localized treatment, minimizing these risks.

PDE4 inhibitors in autoimmune diseases

While crisaborole’s application in AA is novel, PDE4 inhibitors have been extensively studied in other autoimmune diseases, such as psoriasis and rheumatoid arthritis. These studies have demonstrated their ability to modulate inflammatory responses and improve disease outcomes. In the context of AA, a recent review by You et al. highlighted the potential of PDE4 inhibitors in reducing the inflammatory cytokine profiles typical of AA and promoting hair regrowth [21]. Thus, PDE4 inhibitors such as crisaborole reduce inflammation by inhibiting the degradation of cyclic AMP, thereby suppressing the production of pro-inflammatory cytokines, such as IL-17 and TNF-α, which are implicated in AA's autoimmune pathogenesis. This anti-inflammatory effect may mitigate immune dysregulation, reduce perifollicular inflammation, and promote hair follicle recovery. Understanding the mechanisms of PDE4 inhibitors is crucial for their application in other autoimmune diseases, as it allows for targeted modulation of inflammatory pathways common across conditions such as psoriasis and atopic dermatitis. For instance, inhibiting PDE4 reduces T-cell-mediated inflammation, which is a shared feature in many autoimmune disorders, potentially broadening crisaborole’s therapeutic utility.

Efficacy and safety in pediatric patients

In our case, crisaborole provided an effective and well-tolerated treatment option for AA in a 10-year-old patient. The observed hair regrowth after two weeks of treatment supports the potential of crisaborole as a non-corticosteroidal alternative to corticosteroids in pediatric AA. Furthermore, the lack of reported adverse effects aligns with the favorable safety profile observed in clinical studies of crisaborole for atopic dermatitis. However, given the spontaneous nature of AA and the short follow-up duration, further studies are required to confirm the long-term efficacy and safety of crisaborole in treating AA.

Limitations

This case report has several limitations. The most significant limitation is the lack of long-term follow-up, which restricts the ability to assess the durability of treatment effects. The possibility of spontaneous resolution in AA patients cannot be excluded, as the condition often follows a relapsing and remitting course. Additionally, the absence of high-quality photographic documentation diminishes the strength of the visual evidence. Larger, multicenter studies with extended follow-up periods are necessary to establish definitive conclusions.

Parental concerns and treatment preferences

Parental concerns about the safety of corticosteroids in children were a key factor in the decision to switch to crisaborole. Corticosteroid-based treatments, commonly used for AA, pose significant risks in children under 12, including skin atrophy, telangiectasia, and systemic absorption, leading to growth suppression and adrenal suppression. Long-term use may also increase the risk of infections and delayed wound healing. Given its topical application and minimal systemic absorption, crisaborole offers a safer alternative for managing AA in pediatric patients. The increasing interest in non-corticosteroidal treatments underscores the importance of considering patient and parental preferences when selecting treatment options, especially in the pediatric population. Further research, including randomized controlled trials with larger pediatric cohorts, is needed to confirm crisaborole’s efficacy and safety in AA treatment. Long-term studies should assess the durability of hair regrowth, optimal treatment duration, and potential adverse effects in children, particularly those with comorbidities.

## Conclusions

This case report highlights the potential of crisaborole as an effective treatment for AA in pediatric patients. The observed positive outcome, following the failure of corticosteroid therapy, suggests that PDE4 inhibition may offer a promising alternative for managing AA. However, further research, including larger clinical trials with longer follow-up periods, is needed to validate the efficacy and safety of crisaborole in pediatric AA treatment. The favorable safety profile and non-steroidal nature of crisaborole make it an attractive option, particularly in children who may be at higher risk for corticosteroid side effects.
